# Estimated blood loss in pregnant women with cardiac disease compared with low risk women: a restrospective cohort study

**DOI:** 10.1186/s12884-019-2447-8

**Published:** 2019-09-04

**Authors:** Hsu Phern Chong, James Hodson, Tara J. Selman, Lucy E. Hudsmith, Peter J. Thompson, Rachel Katherine Morris, Sara Thorne

**Affiliations:** 1 Department of Fetal and Maternal Medicine, Birmingham Women’s and Children’s NHS Foundation Trust, Edgbaston, Birmingham, B15 2TG UK; 2 Institute of Translational Medicine, Heritage Building Mindelsohn Way, Birmingham, B15 2TH UK; 3 Department of Cardiology, University Hospitals Birmingham NHS Trust, Edgbaston, Birmingham, B15 2TH UK; 4 Institute of Metabolism and Systems Research, University of Birmingham, Edgbaston, Birmingham, B15 2TT UK; 5 Present address: Department of Fetal and Maternal Medicine, Princess Anne Hospital, Coxford Road, Southampton, SO16 5YA UK

## Abstract

**Background:**

Women with cardiac disease are thought to be at increased risk of post-partum haemorrhage. We sought to assess the estimated blood loss (EBL) in our cohort of women with and without cardiac disease (CD) in a quaternary hospital in the UK. Our population consisted of both congenital and acquired CD; and low risk women who delivered in our unit between 01/01/2012–30/09/2016.

**Methods:**

Data were collected using computerised hospital records. CD was classified according to the modified WHO classification (mWHO). The primary outcome measure was estimated blood loss (mL).

**Results:**

A total of 5413 women with a singleton fetus in the cephalic presentation delivered during the study period (159 women with CD and 5254 controls). In the CD group, active management of the third stage of labour was consistent with that used in low risk women in 98% (152/155) of cases. Multivariable analyses demonstrated no significant difference in EBL between women with CD vs controls. The adjusted average blood losses were 247.2 ml, 241.8 ml and 295.9 ml in the control group, mWHO 1–2 and 3–4, respectively (*p* = 0.165).

**Conclusions:**

Women with CD have comparable EBL to low risk women when management of the active third stage of labour is the same.

## Background

Women with heart disease may tolerate post-partum haemorrhage (PPH) poorly, because of a reduced ability to compensate for rapid changes in circulating volume. Two factors in the intrapartum management of these women may increase their risk of PPH. Firstly, concern about their ability to increase cardiac output during the active second stage of labour has led obstetricians to limit the duration of this stage, resulting in an increased chance of instrumental delivery and PPH. Secondly, because of their cardiovascular effects, reduced doses of oxytocic drugs in the third stage of labour are often used in women with heart disease, increasing the risk of PPH [1, 2]. Oxytocin is a nanopeptide with homology to anti-diuretic hormone. Animal and human models of cardiovascular response to oxytocin have demonstrated a hypotensive effect when intravenous administration occurs too rapidly [3]. In all pregnant women with cardiac disease, the Royal College of Obstetricians and Gynaecologists (RCOG) advocates 5 i.u Syntocinon intramuscularly (im) or 2 i.u. intravenous (iv) over 10 min for active management of the third stage [1]. The European Society for Cardiology (ESC) echoes the RCOG recommendation of using 2 i.u oxytocin iv, but makes no mention of intramuscular oxytocin use [4]. These doses are half of those used in healthy women for active management of the third stage [5].

Cauldwell et al. identified a higher mean blood loss at delivery in 366 nulliparous women with congenital cardiac disease, compared to nulliparous women without cardiac disease. They found that the mean blood loss was 439mLs for spontaneous vaginal delivery, 715 mLs after forceps delivery, 722 mLs after emergency caesarean section (CS) and 854 mLs for those who underwent a general anaesthetic [6]. As no information on uterotonics was provided, it is difficult to assess if the blood loss was a consequence of having heart disease or because of management of the third stage.

The Birmingham Women’s and Children’s Hospital provides a quaternary service to women with cardiac disease in the West Midlands. These are women with congenital and acquired disease, encompassing both structural and rhythm abnormalities, as well as ischaemic heart disease. Our service is multidisciplinary, with maternal fetal medicine specialist obstetricians, cardiologists with expertise in congenital heart disease, obstetric anaesthetists and a specialist obstetric haematologist. Management of the third stage is individualised when making their intrapartum care plan. Our protocol for management of the third stage of labour in women with cardiac disease is identical to that for women with low risk pregnancies, unless there is a specific indication for an alternative regimen. Active management of the third stage in the low risk and cardiac population is described in Table 1. In the event of increasing blood loss, our practice is to administer further uterotonics, in line with RCOG guidance on PPH, with the exception that ergometrine is avoided [7]. We sought to evaluate the impact of our practice on estimated blood loss (EBL) following delivery in this high risk population, and hypothesised that the EBL in women with cardiac disease would be similar to women without cardiac disease, as our management of these two groups is essentially identical [7].

**Table 1:** Dose and route of oxytocin for management of the third stage in women with and without cardiac disease in our unit

Oxytocic agents used for active management of the 3rd stage	Spontaneous vaginal delivery	Non-rotational delivery	Rotational delivery in theatre	Elective or Emergency Caesarean delivery
All women *without* cardiac disease in accordance with NICE guidelines on intrapartum care.	10 i.u. oxytocin, intramuscular (im)		5 i.u. oxytocin intravenous (iv), given as a slow bolus over 3–5 min	
Women *with* cardiac disease except those listed below.	10 i.u. oxytocin, im		5 i.u. oxytocin iv given as a an infusion over 10 min through a pump	
Women with the following cardiac conditions: • Single ventricle • Fontan circulation • Valvar stenosis • Severely impaired ventricular function [< 30% Ejection Fraction (EF)]	Management is individualised with consideration of a 5 iu infusion over 10 min through a pump			

The key difference in the administration of oxytocin in our population of low risk women and women with cardiac disease is that intravenous administration occurs over a 10 min interval instead of a slow bolus over 3–5 min. The dose of oxytocin is the same. This regime differs from the RCOG good practice recommendation which advocates a lower dose of 2 i.u. ocytocin given over 10–20 min at elective CS; and 5 i.u oxytocin intramuscularly, or 2 i.u oxytocin over 10 min for women who have a vaginal delivery

## Methods

This was a retrospective cohort study in a quaternary referral centre. Patients were identified from the cardiac obstetric database. All women with cardiac disease who were seen antenatally and delivered at Birmingham Women’s Hospital from 01/01/2012–30/09/2016 were included. We included all women with a past medical history of cardiac disease, carrying singleton pregnancies and delivering a baby in the cephalic presentation. Cardiac disease was categorised according to the modified World Health Organisation (mWHO) classification of maternal conditions at the start of pregnancy [4]. The comparator group consisted of women without cardiac disease who commenced their labour in the adjacent birth centre in our unit during the same period. The following exclusion criteria were applied to the control group:

- Any medical disease (such as epilepsy, gestational diabetes or diabetes mellitus, cardiac disease, liver or renal disease, hypertension)
- Pre-eclampsia or pregnancy induced hypertension
- Body Mass Index (BMI) > 35 kg/m^2^
- Malposition
- Malplacentation
- Multiple pregnancy
- Estimated gestational age under 37 weeks
- More than two episodes of reduced fetal movements
- Fetal growth restriction
- Estimated fetal weight over 4.5 kg.

For all patients included in the study, data were extracted from the departmental records system for age, BMI, parity, gestation, baby weight and the mode of delivery. Blood loss was visually estimated, and although this approach has limitations [8], it is standard practice globally.

### Statistical methods

Initially, a range of factors were compared across the mWHO grades, with the control group treated as “mWHO 0”. Continuous variables were analysed using Jonckheere-Terpstra tests, to account for the order of the mWHO grades, whilst nominal variables were assessed using Fisher’s exact tests. Analyses were then performed to identify factors associated with blood loss, using Spearman’s rho correlation coefficients for continuous variables and Mann-Whitney tests for nominal variables. The distribution of blood loss was highly skewed, and so was summarised using both geometric means and medians with interquartile ranges (IQRs). A multivariable analysis was then performed to identify factors that were independently associated with blood loss. Blood loss values were log_2_-transformed, prior to the analysis, to reduce the degree of skew in the distribution and improve model fit. The coefficients from the resulting model were then anti-logged, and converted into percentage differences. As such, for continuous variables, the reported values represented the percentage increase in blood loss associated with a unit increase in the factor. For nominal variables, the values represented the percentage difference in blood loss between groups. Adjusted average blood losses in each group were then were then calculated, by evaluating the model at the cohort mean value of continuous variables, and for the proportion of patients in each category of nominal variables. All analyses were performed using IBM SPSS 22 (IBM Corp. Armonk, NY), with *p* < 0.05 deemed to be indicative of statistical significance throughout.

## Results

### Patient demographics

Data were available for a total of 5413 patients who delivered between 01/01/2012–30/09/2016. Of these, 5254 (97.1%) were healthy controls, 105 (1.9%) had heart disease and were mWHO grade 1–2 and 54 (1.0%) had heart disease who were mWHO grade 3–4 (Table 2).

**Table 2:** List of cardiac conditions and mWHO classifications

	mWHO classification				
	1	2	3	4	Total
Congenital					
Marfan or other aortopathy including bicuspid valve aortopathy	–	2	10	1	13
Repaired septal defect (atrial, ventricular, atrioventricular)	2	10	1	–	13
Repaired Tetralogy of Fallot		10	2	–	12
Repaired coarctation of the aorta	–	8	1	–	9
Hypertrophic cardiomyopathy	–	4	4	–	8
Complex disease, biventricular repair	–	5	2	–	7
Systemic right ventricle (congenitally corrected transposition, transposition post atrial switch)	–	–	5	1	6
Bicuspid aortic valve without aortopathy	4	–	–	–	4
Transposition of the great arteries, post arterial switch	–	4	–	–	4
Fontan	–	–	2	–	2
Other^a^	–	5	5	–	10
Total	6	48	32	2	88
Acquired					
Regurgitant valvular lesions	4	13	4	–	21
Arrhythmia (normal echo)	16	5	–	–	21
Stenotic valvular lesions	1	4	8	1	14
Dilated cardiomyopathy or previous peripartum cardiomyopathy or other cardiomyopathy	2	1	6	–	9
Ischaemic heart disease	2	3	1	–	5
Total	25	26	19	1	71

^a^ Includes: cardiac transplant, cardiac trauma, cor triatriaum, dilated pulmonary artery, mechanical valve, unoperated atrial septal defect, bioprosthetic valve, complex cyanotic heart disease

Patient demographics are compared between these groups in Table 3. This found that, as mWHO grade increased, there were significant increases in patient age (*p* = 0.012), BMI (*p* < 0.001) and parity (p < 0.001). Increasing mWHO grade was also associated with significantly shorter gestation (p < 0.001) and, correspondingly, a significantly lower baby weight (p < 0.001). The mode of delivery also differed significantly by mWHO grade (*p* = 0.002). Spontaneous vaginal delivery made up 77.2% of cases in healthy controls compared to 42.6% in WHO grade 3–4, whilst rates of caesarean section increased from 6.9 to 44.4% across the categories.

**Table 3:** Factors associated with mWHO grade

	WHO Grade				
	N	Controls	1–2	3–4	*p*-Value
Age at Delivery	5413	28.5 ± 5.1	29.4 ± 5.9	30.1 ± 5.8	**0.012**
BMI	4579	24.4 (21.9–27.6)	26.2 (22.1–31.5)	27.4 (23.8–32.3)	**< 0.001**
Parity	5384				**< 0.001**
*0*		2584 (49.3%)	32 (33.0%)	21 (43.8%)	
*1*		1569 (29.9%)	35 (36.1%)	9 (18.8%)	
*2*		664 (12.7%)	17 (17.5%)	10 (20.8%)	
*3+*		422 (8.1%)	13 (13.4%)	8 (16.7%)	
Gestation at Delivery (Completed weeks)	5412				**< 0.001** ^**a**^
*< 40 Weeks*		2088 (39.7%)	79 (76.0%)	45 (83.3%)	
*40+ Weeks*		3166 (60.3%)	25 (24.0%)	9 (16.7%)	
Baby Weight (g)	5407	3404 ± 431	3150 ± 641	2741 ± 641	**< 0.001**
Mode of delivery	5413				**0.002** ^**b**^
*Spontaneous Vaginal*		4054 (77.2%)	53 (50.5%)	23 (42.6%)	
*Ventouse*		309 (5.9%)	9 (8.6%)	2 (3.7%)	
*Forceps*		528 (10.0%)	6 (5.7%)	5 (9.3%)	
*Caesarean Section*		363 (6.9%)	37 (35.2%)	24 (44.4%)	
Manual Removal	5413				0.297^**b**^
*No*		5219 (99.3%)	104 (99.0%)	53 (98.1%)	
*Yes*		35 (0.7%)	1 (1.0%)	1 (1.9%)	

Data are reported as mean ± SD, median (IQR), or as N (%), as applicable. *p*-Values are from Jonckheere-Terpstra tests, unless stated otherwise. Bold *p*-values are significant at *p* < 0.05.^**a**^ The number of weeks gestation was analysed as a continuous variable when calculating the *p*-value. ^**b**^Fisher’s exact test

### Indications for operative vaginal delivery and CS in women with cardiac disease

Of the 11 women with cardiac disease who underwent ventouse delivery, the most common indication was a non-reassuring cardiotocograph (CTG), with this occurring in 73% (8/11) of women. In one (9%) woman, a ventouse delivery was performed exclusively for the maternal cardiac condition, whilst, in another woman (9%), the decision was made in light of a combination of non-reassuring CTG and the maternal cardiac condition. One woman (9%) required assisted delivery due to maternal exhaustion alone. Forceps deliveries were performed in 11 of the women in the cardiac diseases group. Of these, 64% (7/11) were due to a non-reassuring CTG or abnormal fetal blood sample (FBS) result, with the remainder (36%, 4/11) being due to slow progress in the second stage (exceeded 60 min of active pushing). Of the 61 women with cardiac disease that underwent caesarean sections, 28% (17/61) were exclusively due to their maternal cardiac condition, 19% (12/61) were undertaken due to prior delivery by CS, and the remaining women had other obstetric reasons for CS (Table 4).

**Table 4:** Indications for Caesarean Section in women with cardiac disease

Indications for Caesarean Section	N (%)
Maternal medical disease	17 (28%)
Previous CS	12 (20%)
Presumed fetal compromise	11 (18%)
Other	6 (10%)
Breech	5 (8%)
Delay 1st stage	5 (8%)
Growth restriction	3 (5%)
Failed induction	2 (3%)

### Blood loss

Across the cohort as a whole, the median blood loss was 250 mL (IQR: 200–350), with a geometric mean of 245.7 mL. Whilst on univariate analysis the EBL was found to be increased with mWHO grade, multivariate analyses showed no difference..

#### Univariable analysis

Blood loss was found to increase significantly with mWHO grade on univariable analysis (Table 5), with geometric means of 244.4 vs. 276.0 vs. 326.4 mL for controls vs. women with the disease who were mWHO 1–2 vs. 3–4 (*p* < 0.001). Blood loss was also found to increase significantly with age at delivery (*p* = 0.016), BMI (*p* = 0.014), gestation (*p* < 0.001) and baby weight (*p* < 0.001), and to decline with parity (*p* < 0.001). A significant difference across the modes of delivery was also detected (*p* < 0.001), with the greatest blood loss in caesarean sections, and manual removal of the placenta (MROP) was also significantly associated with increased blood loss (*p* < 0.001).

**Table 5:** Factors associated with blood loss

	N	Blood Loss (mL)		
		Median (IQR)	Geometric Mean	*p*-Value
mWHO Grade				**< 0.001**
*Controls*	5254	250 (177–350)	244.4	
*1–2*	105	300 (200–500)	276.0	
*3–4*	54	400 (200–500)	326.4	
Age at Delivery				**0.016**
*< 25 Years*	1248	250 (150–350)	240.2	
*25–29 Years*	1831	250 (150–350)	244.1	
*30–34 Years*	1622	250 (200–400)	247.4	
*35+ Years*	712	250 (200–400)	256.0	
BMI				**0.014**
*≤ 25*	2535	250 (200–350)	244.8	
*25–30*	1455	250 (200–400)	253.2	
*> 30*	589	250 (199–400)	251.5	
Parity				**< 0.001**
*0*	2637	300 (200–400)	281.6	
*1*	1613	200 (150–300)	222.4	
*2*	691	200 (150–300)	209.3	
*3+*	443	200 (100–300)	199.3	
Gestation at Delivery (Completed weeks)				**< 0.001**
*< 40 Weeks*	2212	200 (150–300)	226.5	
*40+ Weeks*	3200	250 (200–400)	259.9	
Baby Weight				**< 0.001**
*< 3.0 kg*	949	200 (150–300)	213.6	
*3.0–3.4 kg*	2280	250 (150–350)	237.8	
*3.5–3.9 kg*	1705	250 (200–400)	259.0	
*4.0+ kg*	473	300 (200–450)	314.4	
Mode of Delivery				**< 0.001** ^**a**^
*Spontaneous Vaginal*	4130	200 (150–300)	214.7	
*Ventouse*	320	300 (200–400)	279.7	
*Forceps*	539	350 (250–500)	353.4	
*Caesarean Section*	424	500 (400–700)	521.9	
Manual Removal				**< 0.001** ^**b**^
*No*	5376	250 (193–350)	244.8	
*Yes*	37	400 (250–700)	416.3	

*p*-Values are from Spearman’s rho correlation coefficients on the untransformed factors, unless stated otherwise. Bold *p*-values are significant at *p* < 0.05. ^**a**^Kruskal-Wallis test. ^**b**^Mann-Whitney test

Since caesarean sections had been shown to be associated with high levels of blood loss, and were also more likely to be performed in women with heart disease, a subgroup analysis was performed by the mode of delivery (Table 6). This found no significant difference in the amount of blood lost between the control and heart disease groups within the subgroups of patients undergoing vaginal deliveries (*p* = 0.810) or caesarean sections (*p* = 0.079).

**Table 6:** Subgroup analysis by mode of delivery

	Vaginal^a^				Caesarean Section			
	N	Median (IQR)	Geometric Mean	*p*-Value	N	Median (IQR)	Geometric Mean	*p*-Value
mWHO Grade				0.810				0.079
*Controls*	4891	200 (150–300)	230.6		363	500 (400–700)	535.6	
*1 + 2*	68	200 (150–300)	219.7		37	450 (300–500)	419.5	
*3 + 4*	30	250 (200–400)	234.2		24	500 (400–650)	494.1	

*p*-Values are from Jonckheere-Terpstra tests, and *p*-values are significant at *p* < 0.05. ^a^Includes spontaneous, Ventouse and forceps deliveries

#### Multivariable analysis

To adjust for other potentially confounding factors, a multivariable analysis was performed (Table 7). This model identified increasing age at delivery (*p* = 0.008) and baby weight (*p* < 0.001), parity of 0 (p < 0.001), manual removal (*p* < 0.001) and caesarean section (p < 0.001) to be significant independent predictors of increased blood loss. After accounting for these factors, no significant difference in blood loss between the control group and women with cardiac disease who were mWHO 1–2 or 3–4 was detected (*p* = 0.165), with adjusted average blood losses of 247.2 mL, 241.8 mL and 295.9 mL, respectively.

**Table 7:** Multivariable analysis of blood loss

	Coefficient (95% CI)	*p*-Value
mWHO Grade		0.165
*Controls*	–	–
*1–2*	−2.2% (−13.9, 11.2%)	0.739
*3–4*	19.7% (− 1.2, 45.0%)	0.066
Age at Delivery (per Decade)	4.6% (1.2, 8.2%)	**0.008**
BMI (per 10 kg/m^2^)	1.0% (−2.9, 5.1%)	0.624
Parity		**< 0.001**
*0*	–	–
*1*	−11.0% (−14.6, −7.2%)	**< 0.001**
*2*	−16.9% (−21.4, − 12.3%)	**< 0.001**
*3+*	−19.9% (− 25.0, − 14.5%)	**< 0.001**
Gestation at Delivery (Completed weeks)	−0.1% (− 1.7, 1.5%)	0.889
Baby Weight (per kg)	28.6% (23.4, 34.1%)	**< 0.001**
Mode of Delivery		**< 0.001**
*Spontaneous Vaginal*	–	–
*Ventouse*	23.3% (15.0, 32.3%)	**< 0.001**
*Forceps*	47.5% (39.2, 56.2%)	**< 0.001**
*Caesarean Section*	121.1% (107.4, 135.6%)	**< 0.001**
Manual Removal		**< 0.001**
*No*	–	–
*Yes*	59.1% (30.3, 94.1%)	**< 0.001**

Results are from a multivariable general linear model. Blood loss followed a skewed distribution, and so was log_2_-transformed, before being set as the dependent variable. The resulting coefficients from the model were anti-logged, and converted into percentage differences. As such, for continuous variables, the reported values represent the percentage increase in blood loss associated with the stated increase in the factor whilst, for the nominal variables, the values are the percentage increase in blood loss in the stated group, relative to the reference. After excluding cases with missing data, the final model was based on N = 4573. Bold *p*-values are significant at *p* < 0.05

### Third stage management in women with cardiac disease

Of the women with cardiac disease, 3% (4/159) had a physiological third stage. Alternative regimes to those described in Table 1 were used in 2% (3/159) women [cardiomyopathy (*n* = 2), Repaired Tetralogy of Fallot (*n* = 1)]. Of these three women, only one received a reduced dose of oxytocin (3 i.u iv instead of 5 i.u.). One patient received an infusion of oxytocin (20 units in 20 mLs of saline infused over 20 min) as the only uterotonic. The other patient received 5 i.u. of oxytocin, but this was given over 20 min instead of 10 min. We speculate that the variations in the three women were due to clinician uncertainty, as these women delivered “out of hours”. Only seven women received Syntometrine as the primary uterotonic. In five of these women, this occurred prior to the change in NICE guidelines for intrapartum care in 2014. All other women had 10 i.u. intramuscular or 5 i.u. intravenous oxytocin for active management of the third stage, as described in Table 1, and additional uterotonics as required if there were concerns about on-going blood loss.

### Adverse outcomes in women with cardiac disease

A total of 32 women (20%) received high dependency care as a result of their cardiac disease, which was part of standard care for this population. None of the women with cardiac disease required high dependency care as a result of blood loss. None of the women with cardiac disease in our study required balloon uterine tamponade, B-Lynch sutures or hysterectomy. None of the women with cardiac disease in our study population experienced coagulopathy or organ dysfunction as a result of blood loss. Only three (2%) women with cardiac disease required a blood transfusion of 2 units of packed red cells; blood loss in these women was less than 500 mL. All three had cyanotic heart disease, and were transfused to keep their haemoglobin above 120 g/L in order to optimise oxygen carrying capacity. No adverse cardiovascular events were observed after oxytocin administration, as defined by unplanned admission to HDU or the need for fluid resuscitation.

## Discussion

### Main findings

In our cohort of nulliparous and multiparous women with congenital and acquired cardiac disease, we found that the EBL following delivery was low. After accounting for the differences in patient demographics and modes of delivery in this cohort, EBL was not found to be significantly higher than the contemporaneously managed low risk women without heart disease. Our results indicate that active management of the third stage using a standard uterotonic regime in cardiac women is safe, beneficial and results in an EBL comparable to a low risk population. According to NICE guidelines [5], we advise active management of the third stage for all women, however, we advocate this more strongly for women with cardiac disease because of the risk of cardiovascular instability in the event of a PPH.

In our cohort of women with cardiac disease, blood loss was lower than that described by others. Kaoutrolou-sotiropoulou reported on a mixed population of congenital and acquired cardiac disease and women without cardiac disease [9]. There was no difference in blood loss between women with and without cardiac disease. However, the mean blood loss for both groups was approximately 500 mLs with a standard deviation of approximately 350 mL, compared to an arithmetic mean of 301 mL ± 230 mL in our cohort. As blood loss was not subcategorised according to mode of delivery in their study, it was not possible to ascertain if excess blood loss was associated with mode of delivery. The arithmetic mean EBL for women with cardiac disease in our cohort was also lower than that of a previously published cohort of women with and without congenital heart disease for all modes of delivery [6].

### Strengths

Our study has a number of key strengths. Firstly, the use of computerised hospital records enabled us to obtain accurate information regarding uterotonics used and mode of delivery. We employed a number of cross referencing strategies to capture adverse outcomes, such as unexpected admission to the high dependency unit and blood transfusion rates, and were able to report these for our cardiac cohort.

### Limitations

Our study has a number of limitations. Firstly, we used subjectively estimated blood loss and did not weigh swabs or drapes to measure actual blood loss. Therefore, it is possible that blood loss was under or overestimated. However, given that this practice would also apply to the low risk cohort, the effect of over or underestimating blood loss would be negated. Secondly, we lacked detailed data on anti-coagulant usage in the cardiac disease cohort. Although the recommended advice is to cease low molecular weight heparin (LWMH) use as soon as labour starts, we are unable to provide time intervals between the last dose of administration of LMWH to time of delivery, and whether this may have affected the EBL. However, since there was no difference in blood loss between the control and cardiac groups, it is unlikely that LMWH use had a significant impact on blood loss. In a recent study on women with mechanical heart valves, a population of women thought to be most at risk of bleeding, due to anticoagulation use, the incidence of primary PPH was low (2%) [10]. Thirdly, we did not have detailed information on blood pressure changes following intravenous oxytocin administration. However, there were no unexpected admissions to the high dependency unit, and no patient required non-blood product fluid resuscitation as direct consequence of intravenous or intramuscular oxytocin administration. Finally, our cohort included only three women in the mWHO 4 category, where pregnancy is ordinarily contraindicated. Though these three women did not experience adverse effects following intravenous oxytocin administration, caution is advised in this high risk population due to the potential for acute haemodynamic changes secondary to intravenous oxytocin administration. Care should always be individualized within a multidisciplinary team setting.

There is a paucity of evidence to guide the use of uterotonics in the third stage of labour in women with heart disease. As a result, concerns about the potential cardiovascular effects of oxytocin have led to some units avoiding its use completely [11], or using low dose regimes [6] with parallel increased blood loss. Cauldwell et al. undertook a prospective cohort study in women with congenital and acquired heart disease to evaluate differing regimes of oxytocin in the third stage, and its impact on cardiovascular parameters [2]. In their study, one group of 30 women received 12 mUnits/min for 4 h in accordance with the RCOG recommendations for women with cardiac disease, which is their “standard” practice.^1,2^ Another group of 30 women received 2 i.u. oxytocin given iv over 10 min in addition to their “standard” regime. In the “standard” regime group, the mean EBL was 830 mL + 444 mL, and in the intervention group, the mean EBL was 511 + 328 mL. Even their ‘high’ dose oxytocin regime was lower than the standard dose given to healthy women, and the mean EBL for both groups of women was greater than in our cohort of women with cardiac disease managed with standard uteronic care. Specialized drapes to collect blood loss in the Cauldwell study may have improved the accuracy of blood loss, whereas we employed visual estimation of blood loss, an internationally recognized standard albeit with its limitations. Future studies could employ spectrophotometry, considered the “gold standard” for quantifying blood loss in a research context [8].

It is particularly important to minimise blood loss in women with heart disease: those with a preload dependent circulation or an inability to increase cardiac output are at risk of cardiovascular collapse if PPH occurs, and those who require anticoagulation for their heart disease are at inherently higher risk of bleeding. Thus, meticulous care to minimise blood loss around the time of delivery is important to achieving a good maternal outcome.

## Conclusions

Our study demonstrates that active management of the third stage in line with NICE guidelines for women without cardiac disease may be administered safely to women with a broad spectrum of heart disease. This resulted in a low estimated blood loss that was comparable to low risk women without heart disease. Our population consists of women with heart disease in a very specialised unit that has a range of particularly high risk and complex congenital and acquired heart conditions and is, therefore, not representative of most obstetric units. Nonetheless, our findings suggest that in women with cardiac disease, an infusion of 5 i.u oxytocin over 10 min or 10 i.u. intramuscularly may be administered without clinically relevant adverse effects. In doing so, blood loss after delivery and the consequences of postpartum haemorrhage can be minimised. However, careful cardiovascular monitoring is still advisable in some cases, and care individualised, as there may be some that are unusually intolerant of the cardiovascular effects of oxytocin.

## Data Availability

The datasets generated and/or analysed during the current study are not publicly available due current UK regulations with regards to data protection but are available from the corresponding author on reasonable request.

## Abbreviations

BMI: Body mass index
CTG: Cardiotocograph
EBL: Estimated blood loss
EF: Ejection Fraction
ESC: European Society for Cardiology
IQR: Interquartile range
LMWH: Low molecular weight heparin
mWHO: Modified World Health Organisation
PPH: Postpartum haemorrhage
RCOG: Royal College of Obstetricians and Gynaecologists
